# Construction and characterisation of a structured, tuneable, and transparent 3D culture platform for soil bacteria

**DOI:** 10.1099/mic.0.001429

**Published:** 2024-01-30

**Authors:** Liam M. Rooney, Lionel X. Dupuy, Paul A. Hoskisson, Gail McConnell

**Affiliations:** ^1^​ Strathclyde Institute of Pharmacy and Biomedical Sciences, University of Strathclyde, Glasgow, UK; ^2^​ The James Hutton Institute, Invergowrie, Dundee, DD2 5DA, UK; ^†^​ Present address: Department of Conservation of Natural Resources, Neiker, Basque Institute for Agricultural Research and Development, Derio, Spain; ^‡^​ Present address: Ikerbasque, Basque Foundation for Science, Bilbao, Spain

**Keywords:** bacterial culture, optical microscopy, soil microbiology, bacterial colonisation

## Abstract

We have developed a tuneable workflow for the study of soil microbes in an imitative 3D soil environment that is compatible with routine and advanced optical imaging, is chemically customisable, and is reliably refractive index matched based on the carbon catabolism of the study organism. We demonstrate our transparent soil pipeline with two representative soil organisms, *Bacillus subtilis* and *Streptomyces coelicolor*, and visualise their colonisation behaviours using fluorescence microscopy and mesoscopy. This spatially structured, 3D approach to microbial culture has the potential to further study the behaviour of bacteria in conditions matching their native environment and could be expanded to study microbial interactions, such as competition and warfare.

## Graphical Abstract

The complete workflow can be split into three parts: growth and observation, metabolic profiling to provide a stable refractive index matching solution, and production of the 3D soil environment. The 3D culture scaffold was created by cryomilling Nafion resin pellets and size filtration. Chemical processing altered the surface chemistry of Nafion particles and facilitated nutrient binding by titration of a defined liquid culture medium. Metabolic profiling determined non-metabolisable sugars and provided an inert refractive index matching substrate, which was added to the final nutrient titration. Inoculation and growth of the test strain allowed for downstream assessment of colonisation behaviours and community dynamics *in situ* by, for example, optical microscopy.
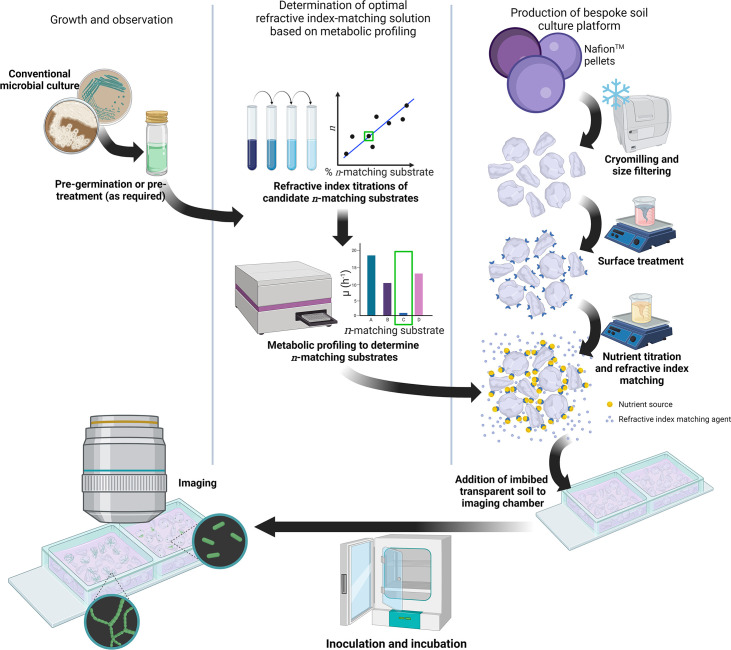

## Data availability

The authors confirm all supporting data and protocols have been provided within the article or through supplementary data files.The datasets generated during and/or analysed during the current study are available from the corresponding author upon reasonable request.

## Introduction

Soil is heterogeneous [1] and its complexity presents a challenge for microbial ecologists attempting to investigate the natural behaviours of microorganisms in a representative culture environment, particularly by means of optical microscopy. Most phenotypic observations of soil bacteria are performed using century-old 2D culture techniques that are unsuitable for *in situ* optical imaging and are neither environmentally nor physiologically representative. The mammalian cell biology field was revolutionised using 3D culture methods over the last 20 years, facilitating discoveries in cell migration and chemotaxis, cell signalling, and tumour development [2–8]. However, these 3D culture methods have been poorly adapted for microbiology applications.

*In situ* microscopy of soil microbes has been impeded due to the incompatible material properties of soil. These include compositional variation [1, 9–11], the presence of bacteriophage and other competing microbes (which can be removed by sterilisation but may also destroy any nutrients) [12], and high autofluorescence and intrinsic scattering which prevents study by many microscopical methods [13]. These drawbacks can be circumvented using transparent refractive index-matched materials to create customisable soil scaffolds, typically achieved by immersing the TS substrate in a liquid medium with the same refractive index of the solid and thereby mitigating reflection and refraction of light through the substrate.

Transparent soil (TS) environments were primarily developed to study plant growth and plant-microbe interactions [14–16], requiring growth media that may have been suboptimal for either co-cultured organism. Moreover, most previous TS platforms have used either no refractive index matching solutions [17] or colloidal silica suspensions to refractive index match the soil substrate [15, 16, 18–20]; however, these suspensions are often unstable over time, resulting in refractive index alterations owed to pH, salinity, and temperature changes.

We present the development of a chemically customisable, microscope-compatible, and environmentally analogous 3D culture method based on a Nafion scaffold, a fluorinated co-polymer of tetrafluoroethylene and perfluorosulfonic acid. We overcome the optical challenges of previous TS applications by using stable and tuneable sugar-based refractive index matching informed by the metabolic profiling of the test organism. Moreover, our workflow provides a flexible solution to facilitate the nutrient requirements of the test strain by permitting titration with various growth media. These two factors present our method as an adaptable technique for the study of microbes in a model 3D environment, especially by optical imaging.

## Methods

The methods below outline our pipeline to construct a tailored 3D culture system for a given test microorganism. These steps involved the creation of a TS scaffold, the methods for which have been adapted from Downie *et al*. [15], and metabolic profiling to determine non-metabolisable sugars for stable refractive index matching.

The methods used to characterise TS in this work are not required to adapt the 3D culture method itself, and so are expanded in Supplementary Methods along with the imaging and bacterial growth methods.

### Production of a Nafion transparent soil scaffold for bespoke culture media

#### Particle size reduction

A 10 g aliquot of Nafion NR-50 1100 EW (Ion Power GmbH, Germany) was cooled by submerging for 5 min in liquid nitrogen before being processed using a cryogenic grinder (6850, SPEX SamplePrep, USA) at the highest frequency (10.0 A.U.) for 2 min. Milled particles were sieved through a series of different pore sizes (500 µm, 850 µm, 1250 µm) (Fisher Scientific, UK) and particles larger than 1250 µm were re-processed as above until they passed through the 1250 µm sieve.

#### Manipulation of Nafion surface chemistry to facilitate nutrient binding

The surface of milled Nafion particles was converted to an anionic form, therefore producing a hydrophilic surface and a negative charge to mimic the water retention profile of soil [15]. This facilitated the downstream binding of nutrient cations. Milled Nafion was immersed in a solution of KOH (15 % w/v), DMSO (35 % v/v) and distilled deionised water (ddH_2_O) (R.2.0/200, Purite Ltd., UK) and incubated at 80 °C for 5 h. The conversion buffer was replaced with ddH_2_O and incubated at room temperature (RT) for 30 min. The particles were washed three times with ddH_2_O and incubated in 15 % (v/v) HNO_3_ at RT for 1 h. Particles were washed three times with ddH_2_O and incubated in 15 % (v/v) HNO_3_ at RT overnight.

Inorganic and organic impurities were removed by washing in ddH_2_O three times and replacing with 1 M H_2_SO_4_. The suspension was incubated at 65 °C for 1 h before cooling down to RT. The H_2_SO_4_ was replaced with ddH_2_O and again incubated at 65 °C for 1 h before cooling to RT. Organic impurities were removed by washing three times with ddH_2_O and submerging in 3 % (w/v) H_2_O_2_ and incubating at 65 °C for 1 h. The suspension was cooled to RT and washed three times with ddH_2_O and stored in ddH_2_O until required.

Prior to nutrient titrations, the TS particles were sterilised by decanting to a flask and submerging with ddH_2_O and autoclaving at 121 °C for 15 min at 15 psi.

### Tailoring transparent soil to produce a bespoke culture medium for *Streptomyces coelicolor*

Streptomycete culture was maintained by titration of TS with Supplemented Minimal Medium (SMM) (2 g l^−1^ casaminoacids, 5.73 g l^−1^ TES buffer [25 mM], 2 ml l^−1^ NH_2_PO_4_ + K_2_HPO_4_ buffer (50 mM of each) [1 mM], 1 ml l^−1^ 1M MgSO_4_·7H_2_O [5 mM], 0.2 ml l^−1^ trace elements solution*, 1000 ml dH_2_O, pH 7.2, *trace elements solution: 0.1 g l^−1^ each of ZnSO_4_·7H_2_O, FeSO_4_·7H_2_O, MnCl_2_·4H_2_O, CaCl_2_·6H_2_O, NaCl. TES buffer: 10 mM tris (pH 7.5), 10 mM ethylenediaminetetraacetic acid (pH 8.0), 0.5 % (w/v) sodium dodecyl sulphate) [21] using 0.2 % (w/v) l-arabinose, a common plant-derived sugar found in soil environments [22], as the carbon source. Processed Nafion particles were titrated by submerging in fresh SMM and shaking for 30 min at 30 °C, 225 r.p.m. The pH was measured after 30 min, and the spent medium was replaced with fresh SMM before again as above. This process was repeated until the pH of the spent medium was equal to that of fresh SMM broth (pH=7.02). SO_3_H^+^ exchange sites were saturated after six titres and the pH stabilised. The particles were then refractive index matched before inoculation (see below). Approximately 1 g of processed TS was placed into a 3 mm deep well of a custom imaging well, as described by McConnell and Amos [23]. A pre-germinated culture of *S. coelicolor* M145 spores was prepared by inoculating 1×10^4^ spores per millilitre into 10 ml of 2xYT medium (16 g tryptone, 10 g yeast extract, 5 g NaCl, 1000 ml dH_2_O) and incubating at 30 °C, 225 r.p.m. for 6 h. The germinated spores were washed twice with sterile TES buffer and suspended in 5 ml of sterile SMM, supplemented with the appropriate refractive index agent (see below). Then 1000 µl of germinated spores in SMM were added to the imaging chamber filled with TS to fully submerge the soil. The lid of a 35 mm diameter plastic dish (81158; ibidi GmbH, Germany) was placed over the well and the imaging chamber was placed in a square 100 mm dish (267060; ThermoFisher, USA) with dampened paper towels to preserve the humidity during incubation. The culture was incubated at 30 °C for 5 days, after which the soil remained saturated with liquid medium and was ready for imaging.

### Tailoring transparent soil to produce a bespoke culture medium for *Bacillus subtilis*

The above method of nutrient titration was adapted for *B. subtilis* culture using Spizizen Minimal (SM) medium [24] with d-glucose used as the sole carbon source (2 g (NH_4_)_2_SO_4_, 14 g K_2_HPO_4_, 6 g KH_2_PO_4_, 1 g Na_3_-Citrate·2H_2_O, 200 mg MgSO_4_·7H_2_O, 500 mg tryptophan, 5 ml 50 % (w/v) d-glucose, 1000 ml dH2O. SO_3_H^+^ exchange sites were saturated typically after six cycles once the pH stabilised (i.e. at pH 7.00). The particles were then refractive index matched (see below) before inoculation with a diluted mid-log phase culture of *B. subtilis* 168, which was washed twice using sterile SM medium. Approximately 1 g of processed TS was placed in an imaging chamber as described in the paragraph above and submerged with 1000 µl of SM medium supplemented with the appropriate refractive index matching agent and a 20 µl of mid-log phase culture. The culture was incubated as described above 30 °C for 2 days, after which the soil remained saturated with liquid medium and was ready for imaging.

### Metabolic profiling to identify non-metabolisable sugars for refractive index matching

Logan and Berkley showed that *B. subtilis* is unable to metabolise certain carbon sources, but the specific strain studied was unclear [25]. We performed a targeted metabolic screen to determine the carbon utilisation of *B. subtilis* 168 strains for refractive index matching candidates. Four sugars were selected from Logan and Berkely’s original screening, d-glucose, d-sorbitol, d-xylose and d-arabinose, and supplemented to SM medium (final [0.2 % (w/v)]). A positive control of LB (Lennox) medium was used.

A 96-well optical bottom plate (265 300, ThermoFisher Scientific, USA) was prepared with 198 µl of either SM medium supplemented with a test carbon source, or LB (with selective antibiotics where required). Overnight cultures *B. subtilis* 168 and JWV042 (a fluorescent derivative strain) were grown in LB broth before diluting in fresh LB broth and growing to OD_600_=0.5. The cells were washed twice with sterile 1 x SM buffer to remove any nutrient carryover. Two micro-litres of the washed cell suspensions were added to the respective wells before the plate was loaded into a Synergy HTX multi-mode plate reader (BioTek Instruments Inc., US) set to medium orbital shaking at 30 °C while measuring OD_600_ every 15 min for 24 h. Blanks containing sterile SM medium with each carbon source and sterile LB were also included. The specific growth rate (μ *h^−1^*) was calculated to quantify the growth on different nutrient sources and empirically identify non-metabolisable sugars, as described previously using Equation 1, where; *N*=the number of cells (OD_600_) at the final measurement timepoint (*t*) and *N_0_* =number of cells (OD_600_) at initial measurement timepoint (*t_0_*).

Eq. 1 $$\mu =\;\frac{\left(\log_{10}N-\log_{10}N_{0}\right)\cdot 2.303}{t-t_{0}}$$

Although the method proposed was tailored to *B. subtilis*, a similar approach could be used to design the RI matching solution for other microorganisms.

*Streptomyces coelicolor* M145 is well-documented in its inability to metabolise sucrose [26, 27], which was verified by assessing the central carbon metabolism using the Kyoto Encyclopaedia of Genes and Genomes [28].

### Refractive index measurements of Nafion and refractive index matching candidates

The refractive index of naïve Nafion particles was measured using Jamin-Lebedeff interferometry (Supplementary Methods), and the measured value was used as a target for candidate refractive index matching solutions. The refractive index of matching solutions was measured using an Abbe refractometer (Billingham and Stanley Ltd., UK) calibrated with methanol at 21 °C (λ=589 nm). A standard curve of concentration versus refractive index was prepared for each candidate in triplicate. Percoll was measured from 1–100 % (v/v) diluted in distilled water (dH_2_O), sucrose was measured from 1–100 % (w/v) diluted in SMM, d-xylose and d-arabinose were both measured from 1–50 % (w/v) diluted in SM medium. Linear regression determined the concentration of refractive index matching agent to be supplemented into the final 3D growth medium to refractive index match Nafion.

## Results

### Characterisation of Nafion-based transparent soil for a customisable microbial culture platform

**Fig. 1.**
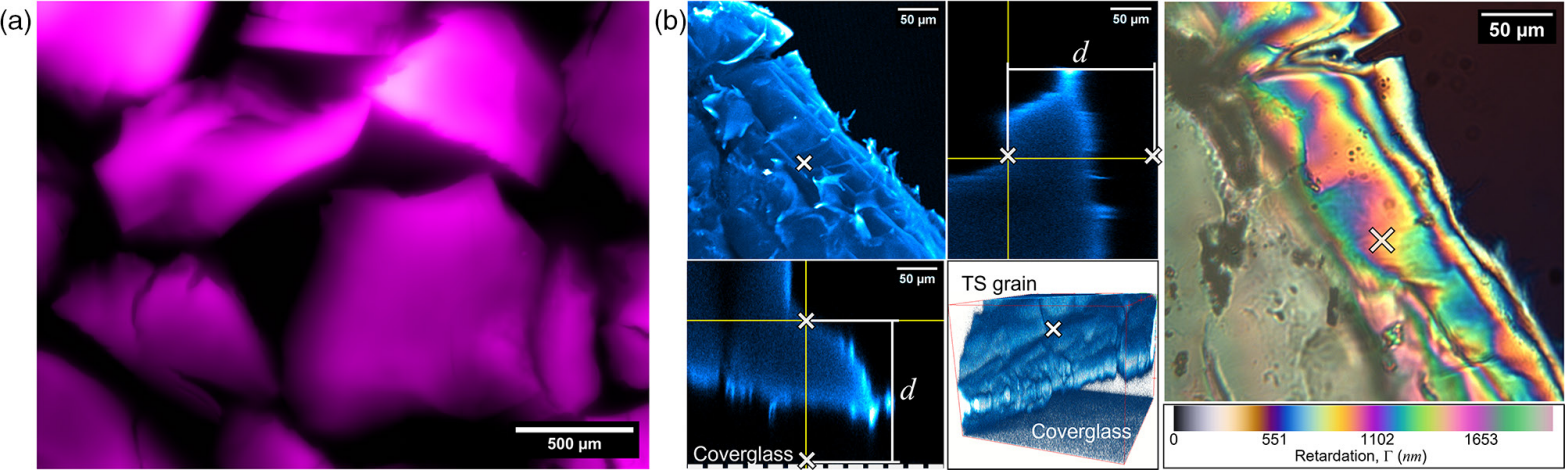
Optical characterisation of Nafion-based transparent soil. (a) A widefield epifluorescence image showing a representative batch of cryomilled Nafion prior to chemical processing. The particles have been stained with sulforhodamine-B and false-coloured in magenta. (b) The refractive index of cryomilled Nafion was measured using Jamin-Lebedeff interferometry. A confocal *z*-stack maximum intensity projection, orthoview, and 3D reconstruction (cyan) provided the depth (d) of the measurement region (denoted by the distance between the white crosses in the orthogonal views, which reflects the position of the flat region noted on the intensity projection and 3D reconstruction). The Jamin-Lebedeff image of the Nafion particle exhibited several interference orders which corresponded to the Michel-Lévy scale (bottom). The phase retardation at the flat measurement region noted by the white cross was Γ = 1400 nm.

The optical properties of chemically processed Nafion TS were verified before developing a bespoke culture platform (Fig. 1). Freeze-fractured, surface-modified, and sulforhodamine-B-stained TS was imaged to verify the size/shape distribution of TS particles (Fig. 1). All particles measured 500 µm to 1250 µm in diameter, with irregularly shaped edges, as desired and in agreement with previous TS applications [15, 16, 18–20]. The refractive index of processed TS particles was calculated using the phase information acquired with a Jamin-Lebedeff interferometer (Figs 1 and S1, available in the online version of this article). The depth (*d*) of the measurement region (white cross in Fig. 1), chosen due to its relative flatness and therein more reliable measurement, was 210 µm. The phase retardation (Γ) at the measurement region was approx. 1400 nm. The refractive index was calculated using the above values and the known refractive index of the mounting medium, distilled water (*n_1_* =1.333), resulting in the refractive index of Nafion TS to be approx. 1.340, which concurs with measurements of other Nafion polymers [29].

### Exploiting non-metabolisable sugars for stable, inexpensive, and accessible refractive index matching

The conventional approach to refractive index matching in TS systems is by supplementation of colloidal silica. Commercial silica preparations can destabilise over long periods or from environmental changes, which can be caused by microbial metabolism. A more stable alternative is required for refractive index matching.

**Fig. 2.**
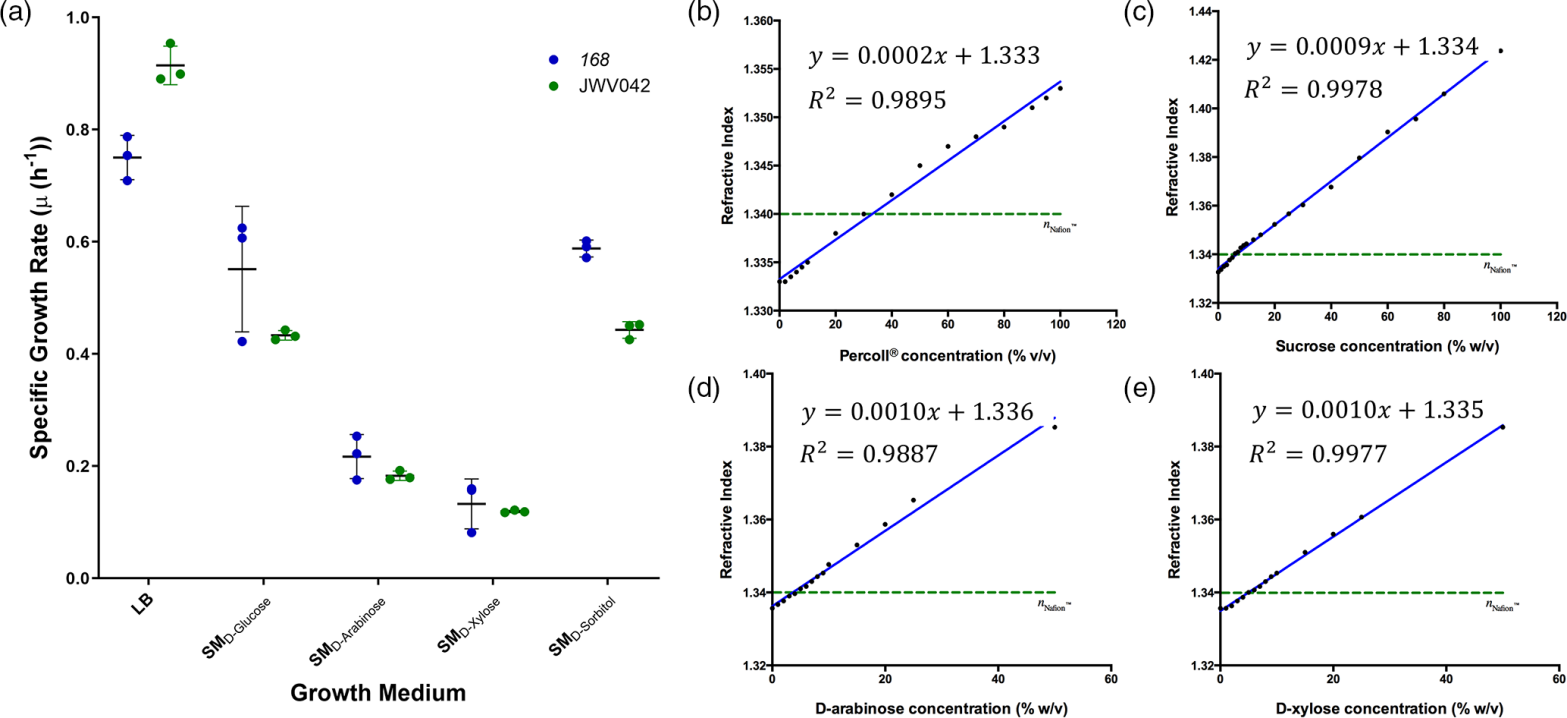
Identification of the optimal refractive index matching sugar by metabolic profiling. (a) The specific growth rate (µ (h^-1^)) of *B. subtilis* 168 and JWV042 in different minimal media containing different sole carbon sources compared with growth in LB. Strains in LB had significantly higher growth rates compared to incubation in minimal media. SM_d-arabinose_ or SM_d-xylose_ exhibited minimal growth, while SM_d-glucose_ or SM_d-sorbitol_ resulted in approximately 50 % reduction in growth compared to LB. (b) A standard curve measuring the refractive index of Percoll dissolved in distilled water. The required concentration to refractive index match Nafion was 35 % (w/v) for Percoll, (c) 6 % (w/v) for sucrose, (d) 3.8 % (w/v) for d-arabinose, (e) 5.1 % (w/v) for d-xylose. Blue fit = standard curve, green line = refractive index for Nafion.

Carbon catabolism has been well studied in many bacterial species and provided a starting point to determine the optimal non-metabolisable sugar for TS refractive index matching. We compared their growth in LB medium to SM medium, with each sugar as a sole carbon source. The specific growth rate was calculated; *B. subtilis* 168 and JWV042 were unable to metabolise d-arabinose and d-xylose (Figs 2a and S2). The small growth rates observed were consistent with measurement errors over time compounded by the build-up of condensation on the lid of the plate.

Our other test organism, *S. coelicolor* M145 and its derivatives have a well-documented inability to degrade sucrose [26, 27]. Therefore, we reliably proceeded with sucrose to refractive index match any streptomycete experiments.

A standard curve of Percoll, a commonly used refractive index-matching medium for TS, was set up to measure the refractive index using an Abbe refractometer (Fig. 2), and it was determined that a 35 % (v/v) Percoll solution was required to match Nafion.

We determined the concentration of each sugar required to refractive-index match Nafion TS by measuring the refractive index of the three identified non-metabolisable sugars. Standard curves of sucrose, d-arabinose, and d-xylose (Fig. 2) revealed that 6.6 % (w/v) sucrose was required to refractive index match TS for *S. coelicolor* observations and that 3.8 % (w/v) d-arabinose or 5.1 % (w/v) d-xylose was required for *B. subtilis* observations.

### Implementation of bespoke 3D culture platform to image the colonisation behaviour of *B. subtilis* and *S. coelicolor*

Following the identification of a suitable non-metabolisable sugar for refractive index matching, the 3D culture system could then be tailored for the study of individual strains of bacteria. We selected two phylogenetically distinct soil organisms to demonstrate the applications of this method, *B. subtilis* and *S. coelicolor*. A defined liquid growth medium was selected for each strain to maintain low levels of autofluorescence that would not prohibit downstream applications involving fluorescence. The nutrient-rich medium, Yeast Extract Malt Extract (YEME), was initially selected for streptomycete culture in transparent soil. However, YEME quickly turned transparent after the first addition and the Nafion particles were dyed yellow, indicating that medium components had saturated the surface of the particles.

**Fig. 3.**
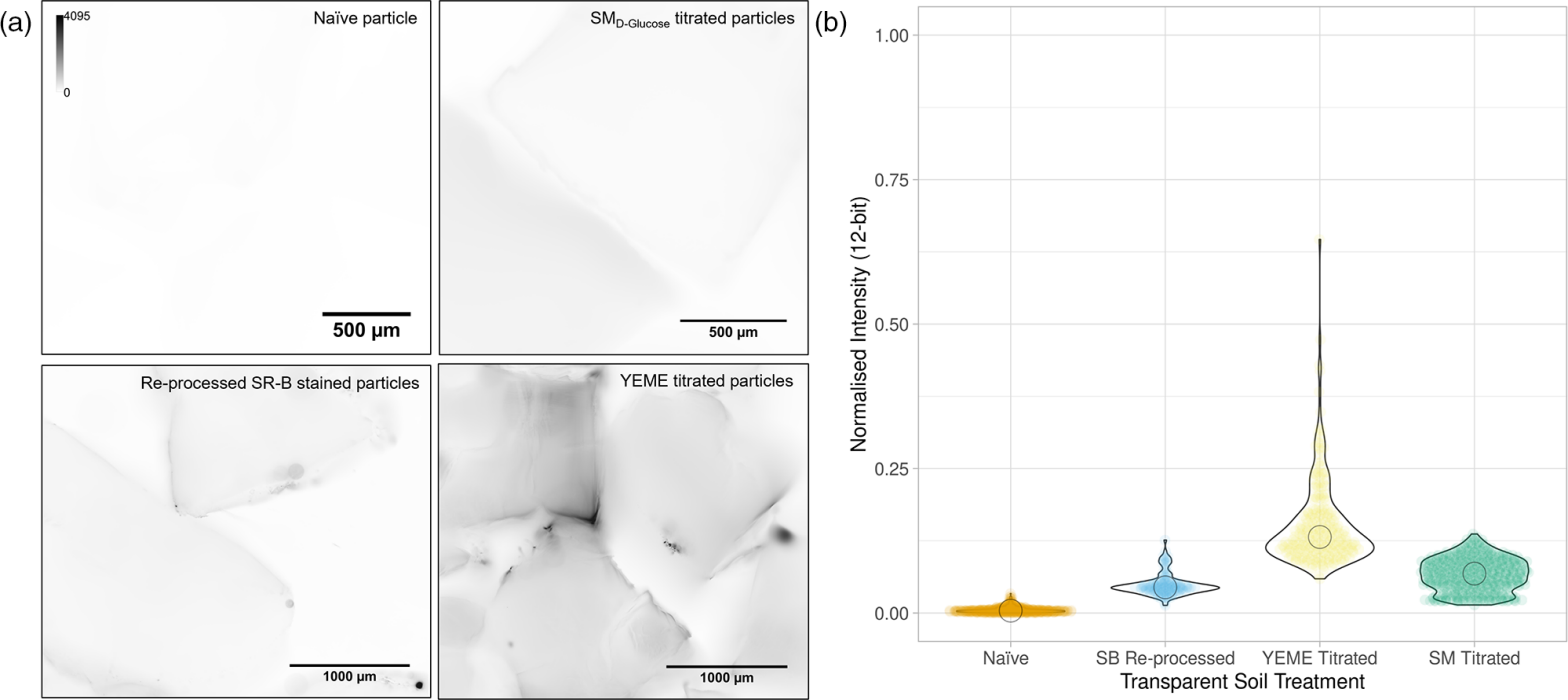
Autofluorescence of Nafion-based TS depends on the components of the growth medium. (a) Mesolens images of chemically processed Nafion particles acquired using 490 nm excitation. Particles tested were naïve, recycled after being stained with sulforhodamine-B, titrated with SM _d_ _-glucose_ medium, or titrated with YEME medium. The intensity scale was inverted to highlight autofluorescence for presentation. (b) Quantification of the autofluorescence signal from each TS specimen is shown in (a), where YEME-titrated TS had the highest autofluorescence intensity.

As our endpoint involved imaging using the Mesolens [30], we first measured the autofluorescence intensity of Nafion TS using the Mesolens (Figs 3 and S3), but also conducted measurements using a standard confocal laser scanning microscope (Fig. S4). Mesoscopic imaging revealed that chemically processed Nafion alone (‘naïve’) had low basal autofluorescence, while TS imbibed with media containing yeast extract, YEME, exhibited 28-fold higher autofluorescence intensity. Autofluorescence intensity from SM_d-glucose_ was 43.23 % lower than that of TS titrated with yeast extract-based media. Autofluorescence measurements of the same particle treatments using a routine confocal microscope revealed negligible autofluorescence signals for all treatments, meaning that more complex media could be explored for routine microscopy applications. The reusability of the 3D culture platform was tested by processing TS particles that had previously been stained with sulforhodamine-B. The dye was sufficiently removed following reprocessing of the stained Nafion and recycled TS had similar autofluorescence levels to naïve TS. Overall, results suggest that the use of media lacking yeast extracts is the best practice to minimise autofluorescence for downstream optical imaging applications and that our 3D culture platform can be repurposed for multiple experiments.

**Fig. 4.**
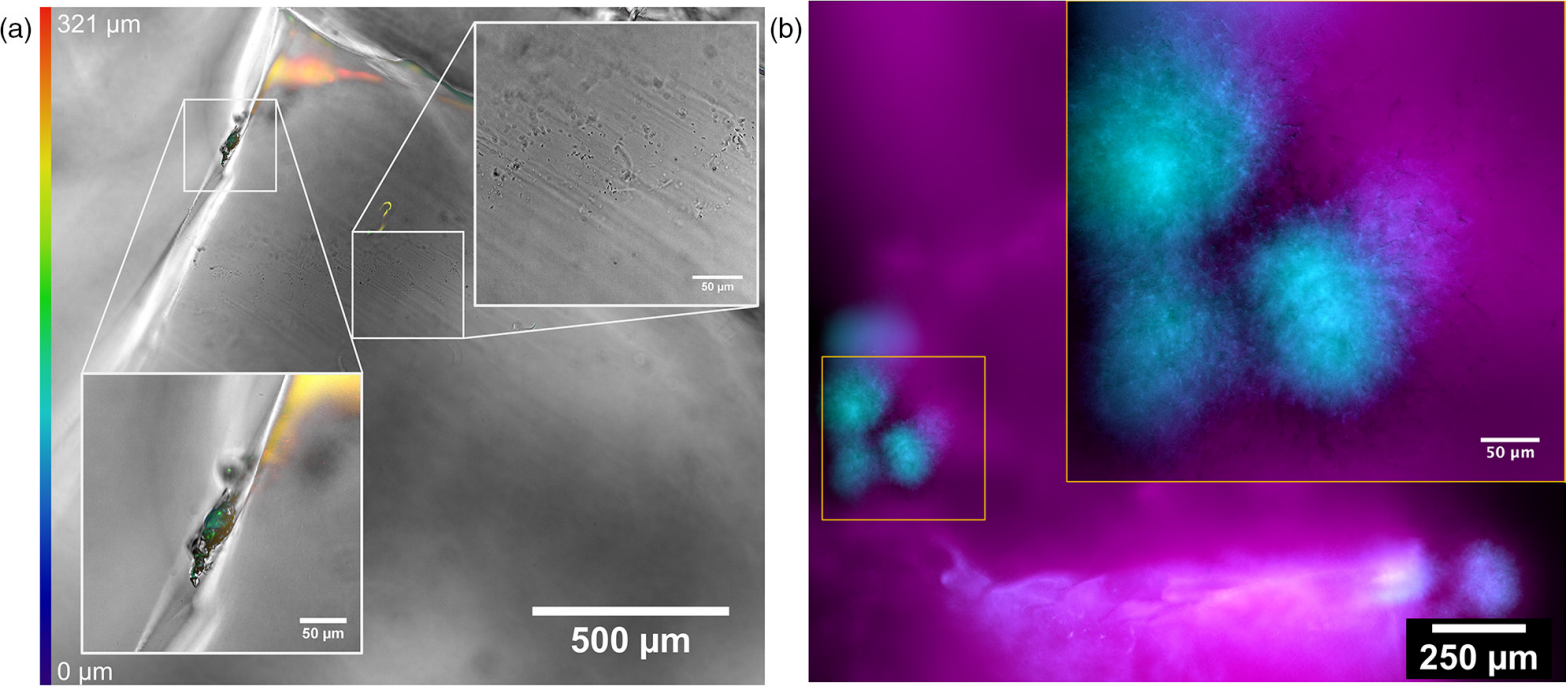
Growth behaviours of soil microbes in a transparent 3D culture environment. (a) A merged widefield epifluorescence and brightfield transmission Mesolens showing colonisation of *B. subtilis*. A *z*-coded colour table was used to show the 3D colonisation of the cells over the entire surface of the particles. Cells either grew as a uniform sparse covering or in discrete biofilms. (b) A widefield epifluorescence Mesolens image *S. coelicolor* grew as isolated microcolonies in protected regions in or between soil particles (*S. coelicolor* idh::gfp = cyan, TS autofluorescence = magenta).

With an inert refractive index matching solution and the requirement for a defined liquid medium identified, SM-titrated TS matched with 3.8 % d-arabinose was selected for *B. subtilis* imaging and SMM-titrated TS matched with 6.6 % sucrose was used for *S. coelicolor* imaging. The application was demonstrated with cultures incubated in their respective tailored 3D culture medium and imaged using either a routine confocal laser scanning microscopy or the Mesolens setup in widefield epifluorescence or transmission brightfield mode. We demonstrated the application of our customisable 3D culture platform to study the colonisation behaviours of *B. subtilis* and *S. coelicolor* (Fig. 4) in comparison to their routine growth behaviours under traditional laboratory culture condition (Fig. S5).

*Bacillus subtilis* exhibited two distinct colonisation behaviours when cultured in transparent soil. Fig. 4 shows a homogeneous but sparse colonisation of individual cells over the entire surface of TS grains in 3D and the presence of isolated biofilms in protected sites, such as grooves and cracks on the TS particle surface. *Streptomyces coelicolor* displayed one colonisation behaviour (Fig. 4). Dense microcolonies formed in protected regions, with hyphae extending from a dense mycelial cluster.

The prolonged viability of bacteria was tested by recovering *B. subtilis* JWV042 cells following 7 days of incubation in a 3D culture scaffold. Cells were recovered and grown on rich medium and observed by widefield epifluorescence microscopy to ensure that cells remained viable during culture in the TS system (Fig. S6). Cells were easily recovered, regrown, and displayed the same phenotype as their initial inoculum, showing that our culture platform could maintain cell viability over multiple days.

## Discussion

We report a methods pipeline to create a customisable, microscope-compatible, and environmentally imitative 3D culture platform for the growth and observation of soil microbes. Current transparent soil techniques are based on a limited set of materials because of the combined requirement for low refractive index and transparency. Two materials have been tested in microbial studies, namely Nafion [15, 16] and cryolite [17], but fluorinated ethylene propylene (FEP) [31] and hydrogels [32] have been tested in root studies and have potential for application in microbiology. Previous applications of transparent soil methods have been demonstrated in plant science [15, 16, 18, 19] and interkingdom co-culture [17, 20]. Nafion is the most suitable platform for microbial studies. Unlike cryolite, it is commonly available commercially and extremely mechanically and chemically stable. Hydrogel alternatives are typically soft and their functionalised polymeric structure is likely to interact with many microbial processes. However, Nafion granules can easily be processed to a desired porosity using cryogenic milling techniques (the limitation to low porosity and small particle sizes is linked to the reduction of the hydraulic conductivity and the time required to prepare samples and process used TS, and the presence of air trapped). Nafion precursor can be tailored to exchange either cations or anions [15]. Because it possesses strong ionic strength, controlled titration with anions and cations can result in soils of a broad range of pH. Recent developments showed FEP is another promising fluorinated polymer. It was used to combine light sheet imaging and optochemical sensors to image pH gradients induced by root exudation in unsaturated conditions [31]. However, FEP requires more advanced chemistry to modify its surface properties.

To date, limitations of TS include maintaining optical transparency, sub-optimal bacterial growth media, and the uncharacterised optical properties of TS. Our method optimises the transparency of the scaffold, manages autofluorescence, and provides scope for application to the culture of other soil microbes, allowing for *in situ* observation of colonisation dynamics and behaviours that may be impossible to replicate with current 2D laboratory culture methods.

We performed robust optical characterisation of our TS system and provide means to implement a custom refractive index matching process to render the substrate transparent. We demonstrated its use by culturing two phylogenetically distinct soil bacteria and present this as a flexible method for the soil microbiologist to adapt to use with their study organisms. Our pipeline is easily adaptable for the microbiologist to transfer to their test organism of choice and has the potential for future applications using fastidious isolates, investigating polymicrobial interactions, phenotyping of monocultured populations in representative environmental conditions, and mimicking extreme soil environments.

We first characterised the optical properties of Nafion transparent soil to inform the downstream chemical processing and refractive index matching requirements. It is important to note that optical characterisation is not routinely required for adapting our method by other users. Users may use our calculated values to optimise their own set-up. A refractive index of 1.340 for Nafion TS was calculated using Jamin-Lebedeff interferometry, agreeing with other measurements of bulk Nafion [29]. Jamin-Lebedeff interferometry was used as it provided enhanced sensitivity for refractive index measurements of soil substrates over, for example, Abbe refractometry which is typically used for liquid samples [33].

Nafion was selected due to its commercial availability, documented use for bioimaging, stability during autoclave sterilisation, low refractive index, potential for particle size refinement, and ion exchange capacity for nutrient imbibement [15, 34]. Using Nafion surmounted the optical and material complexities and costs of previously published artificial soil scaffolds such as those using cryolite [17, 35], FEP [36], silica [37, 38], and hydrogels [32, 39]. The material properties of Nafion permitted particle size refinement from centimetre to micron-scale particles. The robustness of Nafion ensured that particles remained of equivalent size after each milling step and provide a reliable pore size and packing, whereas more brittle substrates would be prone to complete trituration. Moreover, the controlled milling process can allow for the reconstitution of different grain sizes to better represent the diversity of soil particle size distribution in a given environment. The ability to chemically modify the surface residues of Nafion particles provides scope to alter the chemical properties of artificial microcosms. We demonstrated that our platform was compatible with conventional fluorescence microscopy and applied the Mesolens with our culture method to take advantage of the high spatial resolution over a large field of view [30].

Following optical characterisation, we demonstrated a straightforward pipeline for stable sugar-based refractive index matching. This overcame the transiency of colloidal silica matching solutions in mutable environments and provided an accessible method for refractive index matching. Recently, transparent soil microcosms were developed to study soil microbes using cryolite and Nafion slurries [17]. However, this application did not use optimal refractive index matching for high-quality optical imaging, negating the potential for longitudinal study or high-resolution optical imaging.

Commonly available sugars were prime candidates for refractive index matching agents due to their stability and high refractive index in solution. However, it was imperative that the study organism could not metabolise said sugar, otherwise, the refractive index of the culture medium would vary over the growth period. The use of sugars as refractive index matching agents provided the advantage of increased stability over time, less sensitivity to the media changing pH or temperature during incubation, and a higher degree of flexibility to refractive index tuning due to the inherently high refractive index of sugars. Moreover, the sugar concentrations we used were consistent with routine culture media recipes and low enough to not introduce osmotic pressures on growing cells [26, 27]. Furthermore, sugars represent a significant financial benefit as opposed to colloidal silica suspension; for example, the use of sucrose saw a cost reduction of 97 % compared to using Percoll (£2.00 per litre 6.6 % (w/v) sucrose versus £868.00 per litre 35 % Percoll, at the time of writing). It is important to note for future applications which study more complex communities, which may utilise different sugars, that alternative matching could be achieved using alternative non-degradable substrates, such as iodixanol.

Autofluorescence was visualised using conventional confocal microscopy and mesoscopy. The detected autofluorescence was negligible using a routine fluorescence microscope that would be commonly available to other users. We then quantified the autofluorescence using the Mesolens to provide insight into the effects of different base nutrient solutions. The Mesolens was chosen for these measurements due to its 25-fold enhanced collection efficiency over a conventional optical microscope of the same magnification; this is owed to the unique ratio of magnification to numerical aperture, which also provides high spatial resolution throughout a large imaging volume [30]. Our data showed that imbibing media containing yeast extract increased the detected levels of autofluorescence from the TS substrate. However, depending on the catabolism of the study organism, autofluorescence could be diminished over time as microbes metabolise autofluorescent nutrient sources. Therefore, to provide maximum fluorescence imaging quality, users are advised to use minimal media which avoids the use of yeast extract components. Minimal media recipes such as these have been suggested to be more akin to soil conditions, making the platform more representative of environmental conditions. Moreover, we demonstrated that used TS preparations can be reprocessed and recycled so that the substrate may be reused.

We applied our tailored culture medium to two distinct soil microbe monocultures and observed their colonisation behaviours using fluorescence microscopy and mesoscopy. *Bacillus subtilis* and *S. coelicolor* were selected owing to their status as model organisms in soil ecology and secondary metabolite production. *Bacillus subtilis* routinely represents approximately 1.6 % of the bacterial titre of terrestrial soil whereas *Streptomyces* spp. are the dominant genus of Actinobacteria, representing 4.7 % of soil bacteria and titres typically ranging from 1×10^6^ to 1×10^9^ colony forming units per gram of soil. The role of *B. subtilis* as an endophyte of plant roots has been well documented and is correlated with increased plant growth, salt tolerance and protection from pathogenic fungi. Moreover, streptomycetes are linked with the production of clinical and agricultural drugs, but the *in situ* biosynthesis of these compounds is unknown in an ecological setting. Some studies suggest that the concentration of bioactive metabolites in the environment is too low to have bactericidal effects, proposing instead that secondary metabolites may instead serve as inter-species signalling molecules between adjacent strains within soil microcosms. The biosynthetic potential and growth behaviours of these organisms has only been explored using 2D conventional culture methods, or almost exclusively in the context of the commensal rhizosphere systems. Moreover, studies have shown that behavioural changes are exhibited by streptomycetes and bacilli when changing from static solid to agitated liquid culture conditions – the 3D transition may be further explored using a 3D culture scaffold. New understanding of 3D growth behaviours may lead to improved conditions to study growth dynamics, ecological modelling, and the elicitation of secondary metabolite production. We observed distinct modes of colonisation in both organisms which verify their previously described environmental life cycles and provide evidence of viability maintenance over 7 days of incubation in our 3D culture medium. Previous studies noted that the culturability of soil microbes could be improved by growing them in mimetic low nutrient structured environments [40]. We have delivered a solution to facilitate these requirements and demonstrated its application. Our application overcomes the limitations of several other methods; the high autofluorescence and opacity of sterilised soil [41–43], the poor spatial resolution of correlative confocal/MALDI-MSI (Matrix-Assisted Laser Desorption/Ionisation-Mass Spectrometry Imaging) [44], and the non-representative spatial structure of fibreglass [45, 46] or poly(ethene glycol)-based hydrogel scaffolds [47].

Adaption of our 3D culture method presents multiple new possibilities for the field microbial imaging. We have demonstrated the use of this platform with phylogenetically distinct soil bacteria and visualised the difference in growth behaviour using traditional culture methods. Our workflow allows users to customise their own 3D culture medium to suit their organism of study. We described how users may determine the optimum refractive index matching medium by conducting or using pre-determined screens to identify non-metabolisable sugars. The current system could be adapted to study the colonisation or growth behaviours of different microbes, to visualise the physiological effects caused by changing environmental conditions, or to determine the effects of chemical elicitation of new behaviours *in situ*. The flexibility of Nafion-based TS presents an adaptable method for the future study of heterogeneous soil environments, such as those with oxygen, temperature, pH and salinity gradients. Transparent soil has the potential to be easily mounted in a variety of imaging chambers and well plates to facilitate these investigations using both microscopy and spectrophotometric approaches. Moreover, the scope of the platform could be expanded to include titration with growth medium produced from soil extracts to generate an improved model environment, or to facilitate the growth of multiple isolates to study competition and growth dynamics.

## Supplementary Data

Supplementary material 1
