# Supplementary material for: Construction and characterisation of a structured, tuneable, and transparent 3D culture platform for soil bacteria
Source: Microbiology (Reading). 2024 Jan 30;170(1):001429. doi: 10.1099/mic.0.001429 (PMC10866023; doi:10.1099/mic.0.001429)
Supplement: Supplementary material 1 [file mic-170-1429-s001.pdf]

Supplementary Material for '*Construction and Characterisation of a Structured, Tuneable, and Transparent 3D Culture Platform for Soil Bacteria*'

Liam M. Rooney[1*], Lionel X. Dupuy[2†], Paul A. Hoskisson[1], Gail McConnell[1]

ORCiD IDs: LMR - 0000-0002-2237-501X, LXD - 0000-0001-5221-9037, PAH - 0000-0003-4332-1640, GM -0000-0002-7213-0686

[1] Strathclyde Institute of Pharmacy and Biomedical Sciences, University of Strathclyde, Glasgow, UK

[2] The James Hutton Institute, Invergowrie, Dundee, DD2 5DA UK

[†] Current affiliation: Department of Conservation of Natural Resources, Neiker, Basque Institute for Agricultural Research and Development, Derio, Spain and Ikerbasque, Basque Foundation for Science, Bilbao, Spain

*Corresponding Author: liam.rooney@strath.ac.uk

1. Supplementary methods

The methods below outline the characterisation of Nafion™-based TS in this work but are not required to adapt the culture platform itself.

1.1 Bacterial strains and general maintenance

Two representative soil bacteria phyla were selected to characterise our optically transparent 3D culture system, Actinobacteria and Firmicutes, and well-studied species were selected from both phyla; *Streptomyces coelicolor* and *Bacillus subtilis*, respectively.

Streptomyces coelicolor (M145) cultures were maintained on solid Mannitol-Soya Flour (MS) medium (20 g/l mannitol, 20 g/l soya flour, 20 g/l agar, 1 l tap water) and liquid cultures were grown in 2x Yeast-Tryptone (2x YT) broth (16 g/l tryptone, 10 g/l yeast extract, 5 g/l NaCl, 1000 ml dH_2O). Yeast Extract Malt Extract (YEME) medium (3 g/l yeast extract, 3 g/l malt extract, 5 g/l peptone, 10 g/l glucose, 1000 ml dH_2O)(49) was used to demonstrate the autofluorescence of Nafion™ imbibed with yeast-based media. The GFP-expression strain, *S. coelicolor* M145 containing *idh*::*gfp* in its native location, integrated on the chromosome and was maintained by supplementing all media with 50 µg/ml apramycin. M145-*idh-gfp*(50) was selected as a test strain for this work as *gfp* was translationally fused to *idh*, which is a highly expressed primary metabolic gene encoding an isocitrate dehydrogenase found throughout the cytosol(51).

Streptomycete culture in TS was supported by titration with Supplemented Minimal Medium (SMM) (2 g/l casaminoacids, 5.73 g/l TES buffer [25 mM], 2 ml/l NH_2PO_4 + K_2HPO_4 buffer (50 mM of each) [1 mM], 1 ml/l 1M $MgSO_4 \cdot 7H_2O$ [5 mM], 2 g/l L-arabinose, 0.2 ml/l trace elements solution*, 1000 ml dH_2O, pH 7.2, *trace elements solution: 0.1 g/l each of $ZnSO_4 \cdot 7H_2O$, $FeSO_4 \cdot 7H_2O$, $MnCl_2 \cdot 4H_2O$, $CaCl_2 \cdot 6H_2O$, NaCl).

Bacillus subtilis 168 and JWV042 cultures were maintained on solid Lysogeny (Lennox) Broth (LB) medium and grown in LB broth (10 g/l tryptone, 5 g/l yeast extract, 5 g/l NaCl, 1000 ml dH_2O). The photoprotein encoding plasmid was maintained in JWV042 by supplementing the growth medium with 5 µg/ml chloramphenicol. JWV042 carried a plasmid which had a translation fusion of *gfp* and *hbs*, a gene encoding for the non-specific DNA-binding protein HBsu, which was under the control of an endogenous promotor (*cat amyE*::P_{hbs}-*hbs-gfp*)(52).

Bacillus culture in TS was supported by titration with Spizizen Minimal (SM) Medium (2 g/l $(NH_4)_2SO_4$, 14 g/l K_2HPO_4, 6 g/l KH_2PO_4, 1 g/l Na_3-Citrate·$2H_2O$, 200 mg/l $MgSO_4·7H_2O$, 500 mg/l tryptophan, 5 ml/l 50% (w/v) D-glucose, 1000 ml dH_2O).

1.2 Fluorescent staining of *S. coelicolor* for routine laboratory growth observation

To visualise the structure of *S. coelicolor* grown under conventional laboratory conditions, specimens were stained using a method known as Schwedock staining(53). Briefly, this method uses two fluorescent dyes, fluorescein isothiocyanate-wheatgerm agglutinin (FITC-WGA) and propidium iodide (PI), to label *N*-acetylglucosamine residues in the peptidoglycan cell wall and nucleic acids, respectively. Cultures were grown on 22 mm x 22 mm type 1.5 coverglass (631-0125, VWR International Ltd., UK) sterilised by immersing in 100% ethanol before passing through a flame. Sterile coverglasses were inserted into solid MS medium at a 45° angle relative to the medium surface. A 5 µl of inoculum of a dilute M145 spore suspension was pipetted on the coverglass-medium interface at the acute angle and incubated at 30°C in darkened conditions for 72 hours (when the erection of aerial hyphae occurred). The coverglasses were aseptically removed from the agar and placed with the mycelium facing upwards abridge two sterile wooden toothpicks in a staining dish. The mycelium was fixed by pipetting 500 µl of ice-cold fixative (2.8% (v/v) formaldehyde (344198, Merck Millipore, USA), 0.0045% (v/v) glutaraldehyde (340855, Sigma Aldrich, USA), pH 7.0) onto the coverglass. The coverglasses were then washed twice with ice-cold phosphate-buffered saline (PBS) (BR0014, ThermoFisher Scientific, USA) before being allowed to air dry. The mycelia were then rehydrated by adding 500 µl of ice-cold PBS and incubating for 5 minutes at room temperature (RT). The PBS was then replaced with 500 µl of ice-cold Glucose:Tris:EDTA buffer supplemented with 2 mg/ml lysozyme (L4631, Sigma Aldrich, USA) and incubated for 1 minute at RT before being washed with ice-cold PBS. Finally, the mycelia were incubated with a blocking solution of 2% (w/v) bovine serum albumin (BSA) (A8531, Sigma Aldrich, USA) in PBS and incubated for 5 minutes at RT.

All subsequent staining steps were performed in darkened conditions. The blocking solution was replaced with 500 µl of an ice-cold staining solution containing 2 µg/ml FITC-WGA (W834, Invitrogen, USA) and 10 µg/ml PI (P1304MP, Invitrogen, USA) suspended in 2% (w/v) BSA in PBS. The coverglass was then incubated with the staining solution for 2 hours at RT. Following incubation, the first staining solution was removed and the coverglass was washed eight times with 500 µl of a second ice-cold

staining solution containing only 10 µg/ml PI in PBS. Before imaging, 10 µl of 40% (v/v) glycerol was used to mount the stained mycelium.

1.3 Characterisation of the optical properties of transparent soil

1.3.1 Sulphorhodamine-B staining of Nafion™ particles

Cryomilled and chemically processed Nafion™ particles were fluorescently stained to visualise the size and shape distribution of particles. 500 µl of 1 µg/ml (1.8 nM) sulphorhodamine-B (S1307, Invitrogen, USA) was added to a sample of milled and chemically processed Nafion™ particles. The dye solution was then removed and placed in a refractive index matching buffer before imaging.

1.3.2 Jamin-Lebedeff interferometry

A Jamin-Lebedeff interferometer (Zeiss, Germany) (Supplementary Figure 1) was used to determine the refractive index of Nafion™ particles. The polarisation optics were aligned before each use to ensure a defined Newton's Series was present at thin regions of the sample. A 10x interference objective, complimentary condenser apparatus, and a C-mounted CMOS colour camera (DKF 33UX264, The Imaging Source, Europe GmbH, Germany) were used to acquire an interference imaging of the Nafion™ particles. White light illumination was sourced from a tungsten lamp. Single grains of cryomilled Nafion™ were mounted between two Type 1.5 coverglasses in dH_2O. A region showing a clear separation of Newton's interference orders was selected, and the colour and order of the measurement region were noted and compared to a Michel-Lévy chart to obtain the phase retardation.

To determine the depth of the sample at the measurement region, the specimen was carefully removed and inverted before placing it on an Olympus IX81 inverted microscope coupled to a FluoView FV1000 confocal scanning unit (Olympus, Japan). The particle autofluorescence was used to gain an overview of the surface in three dimensions. Images were acquired using a 10x 0.4 NA UPLXAPO objective lens (Olympus, Japan) and fluorescence emission was detected using a photomultiplier tube with spectral detection set to 525 nm with a 50 nm bandwidth. A 488 nm line from an Argon laser (GLG3135; Showa Optronics, Japan) was used as an excitation source. A 3D z-stack of the same area imaged using the Jamin-Lebedeff interferometer was acquired at Nyquist rate (Δz = 1.50 µm). The upper and lower limits were used to measure the depth of the measurement region.

The refractive index of Nafion™ (n_2) was calculated using the established relationship between the thickness of the specimen (d), the phase retardation (Γ) at the measurement region, and the known refractive index of the mounting medium (dH_2O) (n_1); $n_2 = {}^{\Gamma}/_{d} + n_1$ (54). For accurate measurement, the mounting medium should be as close to the specimen as possible (i.e., $n_1 = n_2 \pm 0.05$), and so some optimisation is required for specimens of a completely unknown refractive index.

1.4 Imaging conditions

1.4.1 Widefield epifluorescence microscopy

Conventional widefield epifluorescence and phase contrast microscopy were used to compare the cellular morphology and GFP expression before and after the growth of bacteria in a transparent soil environment. Images were acquired using an Eclipse TE-2000-S microscope with a 100x/1.30 NA CFI PLAN FLUO DLL Oil objective lens (Nikon, Japan) coupled to a digital CCD camera (C4742, Hamamatsu, Japan). Illumination for GFP excitation was sourced from a mercury arc lamp which was coupled into the epi-port of the microscope. A 530 nm ± 35 nm emission filter was used for fluorescence imaging (1CH81700, Chroma Technology Corporation, USA). Illumination for phase contrast microscopy was provided by a tungsten bulb light source.

1.4.2 Confocal laser scanning microscopy

A confocal laser scanning microscope was used to verify that no reflection signal was detected from soil particles following refractive index matching and to assess the autofluorescence profile of the transparent soils. Particles were imaged using a Leica TCS-SP5-II confocal laser scanning microscope with a 5x/0.15 NA PL FLUOTAR objective lens (Leica, Germany). Incident light for reflection, autofluorescence excitation and transmission was provided by a 488 nm line from a Kr/Ar laser source (Coherent, USA). Reflection imaging was carried out by placing an 80/20 beam splitter in the detection path and restricting the PMT detection to 488 nm ± 5 nm. The autofluorescence signal was acquired by extending the same PMT detection from 520 nm to 620 nm. Transmission images were acquired by using the TCS-SPT-II in transmission mode and detecting transmitted light using a PMT detector

1.4.3 Widefield mesoscopy

Brightfield transmission mesoscopy was achieved using a tungsten bulb light source and the condenser position, condenser diaphragm, and field iris set for Kohler illumination. Fluorescence excitation was achieved using a 435 nm or 490 nm LED from a pE-4000 LED illuminator (CoolLED, U.K.). A triple bandpass filter which transmitted light at 470 ± 10 nm, 540 ± 10 nm, and 645 ± 50 nm was placed in the detection pathway. The emission signal was detected using a VNP-29MC CCD camera with chip-shifting modality (Vieworks, South Korea) to capture the full FOV of the Mesolens at high resolution. Widefield mesoscopic imaging was carried out using water immersion (n = 1.33) with Mesolens correction collars set accordingly to minimise spherical aberration through refractive index mismatch.

1.5 Image analysis

The autofluorescence of Nafion™ particles following different surface treatments was analysed by first acquiring images of soil particles by widefield epifluorescence mesoscopy. As each test strain of bacteria encoded GFP, the autofluorescence intensity was measured in all samples by exciting with 490 nm LED at moderate power and exposure times (35% LED power, 500 ms exposure time). Autofluorescence intensity was quantified by first drawing multiple ROIs randomly over the surface of transparent soil particles. The mean intensity in each ROI was measured and normalised against the dynamic range of the camera sensor (0 – 4095 AU). The mean values for each surface treatment were then compiled in a violin plot using Plots of Data. Image analysis was performed using FIJI(55). Figures presented were linearly contrast adjusted for presentation purposes where required using FIJI.

1.6 Validating specimen viability in transparent soil media

A phenotypic screening method was used to determine if bacteria remained viable throughout incubation and growth in a transparent soil environment. Following growth and imaging of JWV042 in transparent soil, a 10 µl inoculum was taken from the soil culture and used to inoculate a 5 ml volume of sterile LB broth supplemented with 5 µg/ml chloramphenicol. The culture was then grown overnight at 30°C shaking at 225 rpm before being serial diluted in sterile LB broth to $1x10^{-8}$ and plated on solid LB medium supplemented with 5 µg/ml chloramphenicol and incubated for 18 hours at 30°C in darkened conditions. A sample was also taken from the overnight liquid culture for visualisation under a conventional widefield epifluorescence microscope to establish if cells recovered from transparent soil

remained fluorescent. These cells were compared to cells which had been grown for 48 hours in liquid $SM_{L\text{-}arabinose}$ medium. The cellular morphology was compared using the microscopy data, while colonial morphology was compared using the colonies on the serial dilution plates.

2. Supplementary Figures

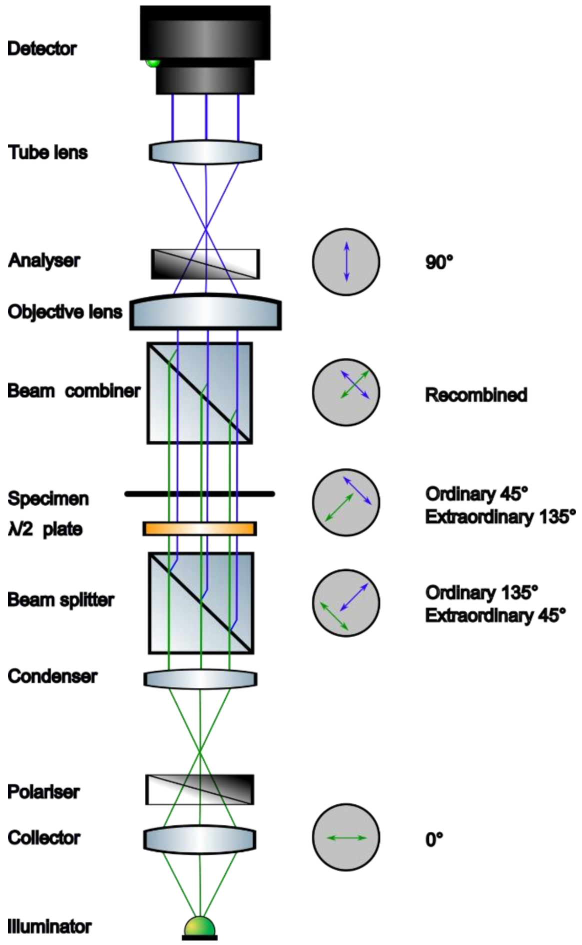

Supplementary Figure 1. Schematic of a Jamin-Lebedeff interferometer. White light illumination is collected and transmitted through a polariser aligned parallel to the optical axis, before being transmitted through a beam splitter which splits the incident light into two paraxial rays: the measurement beam (blue) and the reference beam (green). A rotating half-wavelength plate is incorporated into the condenser apparatus, azimuthally rotating will control the alignment of the Newton's series in the acquired interference image. Light passes through the sample, and the measurement and reference beams will undergo a relative phase retardation depending on their transmittance through a sample of depth, d. The two beams are re-combined by a beam combiner, which rotates azimuthally together with the objective to match the diagonal position of the crossed polariser and analyser. The objective lens then collects the re-combined light to form an image displaying the phase retardation as a coloured Newton's series, as in a Michel-Lévy chart. The analyser is oriented to match the polariser and aligns the plane and axis of the combined beams to cause destructive and constructive interference between the two wavefronts. The light then passes through a tube lens prior to being detected, either by eye or by CCD or CMOS camera.

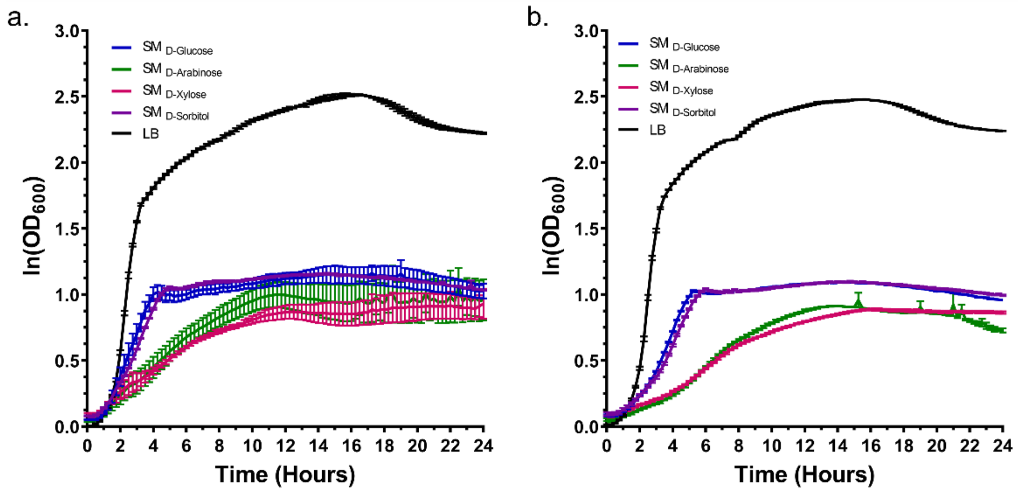

Supplementary Figure 2. Growth curves of *B. subtilis* to determine non-metabolisable sugars. (a) A semi-log growth curve for *B. subtilis* 168 grown on rich medium (LB) and several SM media containing a single sugar carbon source. **(b)** A semi-log growth curve for *B. subtilis* JWV042 grown on rich medium (LB) and several SM media containing a single sugar carbon source.

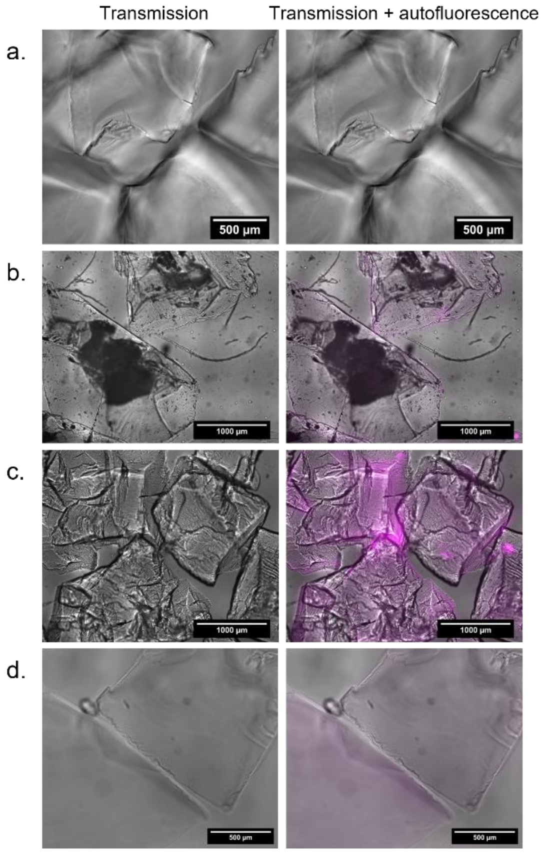

Supplementary Figure 3. Merged brightfield and fluorescence images of TS autofluorescence acquired using the Mesolens. (a) Brightfield transmission (left) and merged fluorescence (right) images of naïve Nafion™-based TS. **(b)** Brightfield transmission and merged fluorescence images of recycled sulphorhodamine-stained TS. **(c)** Brightfield transmission and merged fluorescence images of YEME-titrated TS. **(d)** Brightfield transmission and merged fluorescence images of $SM_{D\text{-}glucose}$-titrated TS. Brightfield transmission is presented in grayscale, autofluorescence is false coloured in magenta. Fluorescence images alone were used to quantify autofluorescence signals in Figure 3.

152

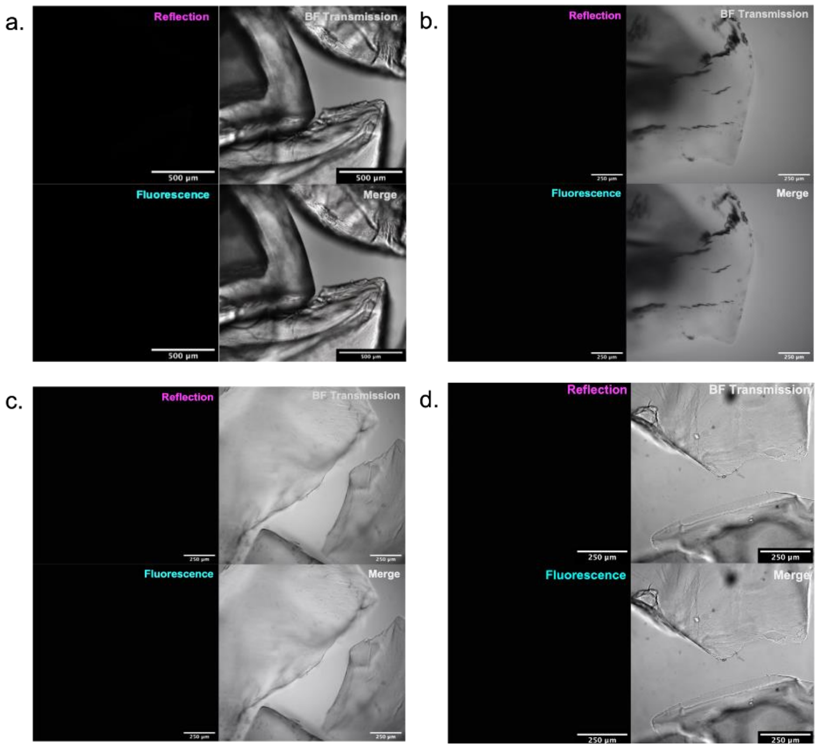

Supplementary Figure 4. Autofluorescence of Nafion™-based TS acquired using a conventional confocal laser scanning microscope. Images acquired using a routine confocal microscope show negligible autofluorescence signal is detected in naïve **(a)**, recycled **(b)**, YEME-titrated **(c)**, or SM-titrated Nafion™ TS **(d).** Brightfield transmission is presented in grayscale, autofluorescence is false coloured in cyan, and reflection is false coloured in magenta.

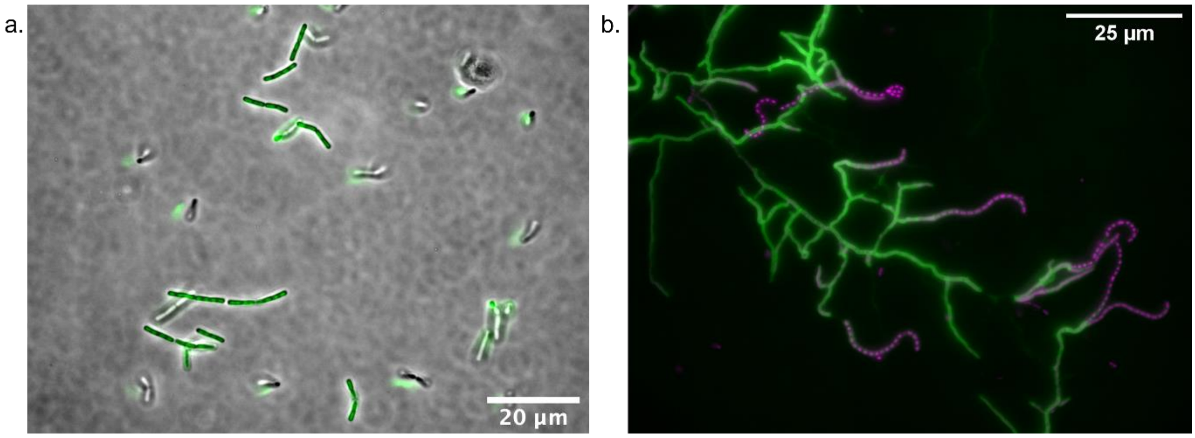

Supplementary Figure 5. Normal laboratory growth behaviours of *B. subtilis* and *S. coelicolor* under traditional culture conditions. (a) *B. subtilis* JWV042 is routinely culture as large multimillimetre colonial biofilms on solid nutrient rich agar or, as shown here, as single cells or short chains in shaking liquid culture. Image shows phase contrast (grey) and Hbs-GFP fluorescence (green) **(b)** *S. coelicolor* routinely grows as a differentiated mycelium, with vegetative hyphae undergoing a series of regulated lifecycle changes to erect aerial hyphae and forming spore chains under nutrient-deprived conditions. Nucleoids are stained with propidium iodide (magenta) and peptidoglycan cell walls are stained with FITC-wheatgerm agglutinin (green).

153

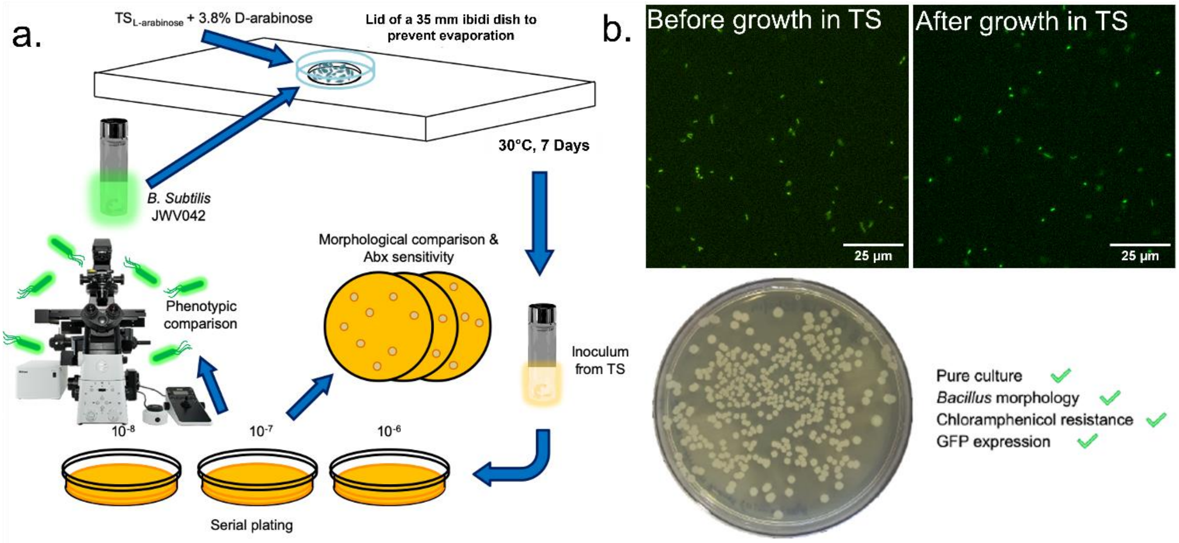

Supplementary Figure 6. Cells remain viable and recoverable after prolonged periods using the tuneable TS culture platform (a) A schematic of the recovery process for *B. subtilis* JWV042 after 7 days of culture in a custom 3D culture medium. Cells were growth for 7 days prior to removal and being serial plated on routine solid culture media. Recovered were grown in the presence of a selective antibiotic and imaged by widefield epifluorescence microscopy to confirm they had the same phenotype as their progenitor inoculum. **(b)** Comparative fluorescence micrographs showing the progenitor and recovered cells after 7 days in 3D culture, Hbs-GFP fluorescence is presented in green. Recovered cell phenotypes were maintained, as shown by plate-based observations.
